# Development, Characterization, and Evaluation of Potential Systemic Toxicity of a Novel Oral Melatonin Formulation

**DOI:** 10.3390/pharmaceutics16070871

**Published:** 2024-06-28

**Authors:** Catalina N. Cheaburu-Yilmaz, Kemal Atmaca, Onur Yilmaz, Hilmi Orhan

**Affiliations:** 1 Biochemistry Division, Department of Chemistry, Faculty of Science, Dokuz Eylul University, 35390 Konak, Izmir, Türkiye; 2 Pharmaceutical Toxicology Department, Faculty of Pharmacy, Ege University, 35040 Bornova, Izmir, Türkiye; ecz.kemal.atmaca@gmail.com; 3 Leather Engineering Department, Faculty of Engineering, Ege University, 35100 Bornova, Izmir, Türkiye; onur.yilmaz@ege.edu.tr; 4 İzmir Biomedicine and Genome Center (İBG-İzmir), Dokuz Eylul University Campus, 35340 Balcova, Izmir, Türkiye

**Keywords:** chitosan coating, itaconic, N-vinyl caprolactam, nanoprecipitation, copolymer, melatonin, biocompatible, systemic acute toxicity

## Abstract

The need to create safe materials for biomedical and pharmaceutical applications has become a significant driving force for the development of new systems. Therefore, a chitosan-coated copolymer of itaconic acid, acrylic acid, and N-vinyl caprolactam (IT-AA-NVC) was prepared by radical polymerization and subsequent coating via nanoprecipitation to give a system capable of sustained delivery of melatonin. Although melatonin brings undoubted benefits to the human body, aspects of the optimal dose, route, and time of administration for the obtaining of suitable treatment outcomes remain under discussion. The entrapment of melatonin in biocompatible polymeric systems can prevent its oxidation, decrease its toxicity, and provide an increased half-life, resulting in an enhanced pharmacokinetic profile with improved patient compliance. The structures of the biopolymer and conjugate were proven by FTIR, thermal properties were tested by DSC, and the morphologies were followed by SEM. The loading efficiency and in vitro release profile were studied by means of HPLC, and a delayed release profile with an initial burst was obtained. The potential systemic toxicity of the formulation was studied in vivo; a mild hepatotoxicity was observed following administration of the melatonin-loaded formulation to mice, both by histopathology and blood clinical biochemistry. Histopathology showed a mild nephrotoxicity as well; however, kidney clinical biochemistry did not support this.

## 1. Introduction

The need to create safe materials for biomedical and pharmaceutical applications has become a significant driving force behind the development of diverse systems. These systems aim to be both biodegradable and non-toxic to the human body while meeting the specific requirements for delivering active substances to their intended targets. Traditionally, polymers were primarily used to mask the unpleasant taste of drugs or as rheological modifiers. However, recent advancements have introduced polymers and polymeric materials with multifunctional capabilities within drug delivery systems. These capabilities include the ability to form films for coating, entrapping, and protecting active substances; gelling properties; biocompatibility; resistance to biochemical degradation; pH- and temperature-controlled characteristics (enabling controlled release and self-assembly behavior), etc.

Among the polymers preferred for their diverse properties, special attention was given to biopolymers, particularly those derived from natural sources (e.g., collagen, chitosan, alginates, hyaluronan, etc.). These biopolymers offer several key advantages, including biological renewability, biodegradability, biocompatibility, relatively low toxicity, and bioadhesive properties, due to their functional groups. Chitosan, for instance, is a linear polysaccharide composed of glucosamine and N-acetylglucosamine units. It stands out as the only cationic pseudo-natural polymer, obtained through the deacetylation of chitin. In the field of pharmaceutical nanotechnology, chitosan has found widespread use as a polymer used for forming particles and, notably, as a surface coating. Thanks to its unique structure, chitosan can impart distinct physicochemical and biological properties to newly developed materials. These properties include enhanced bioadhesion, the capacity to form films on skin and mucous membranes, favorable flow characteristics, and antibacterial activity [1,2,3]. The combination of distinct properties from various biopolymers can result in synergistic qualities in the resulting polymeric material. An intriguing example of an industrial biotechnological compound derived from natural resources is itaconic acid (IT). IT has proven to be a viable option for the synthesis of intelligent polymers [4,5] and has played important roles, such as serving as a biochemical building block, enabling enhancements in structural characteristics [6]. 

Particular attention is directed towards micro/nanoparticles that are constructed from polymers exhibiting a lower critical solution temperature (LCST), one close to the physiological range of 32–38 °C. The widely employed polymer family utilized for crafting responsive nanoparticles is temperature-sensitive poly(alkylacrylamides), with a specific emphasis on poly N-isopropyl acrylamide (PNIPAAm) [2,7,8]. However, due to concerns regarding the toxicity of its monomer, N-isopropyl acrylamide (NIPAM) has recently been avoided in certain applications [9]. Instead, N-vinyl caprolactam (NVCL) is gaining preference due to its biocompatibility, water solubility, and LCST behavior within the physiological temperature range [10,11,12]. This combination of properties allows it to be considered as an adequate material in the design of biomedical devices and useful in drug delivery systems. As proposed within the current study, given the combined properties of itaconic acid, namely, its pH responsiveness, dual functionality, and ability to polymerize with N-vinyl caprolactam, along with its temperature sensitivity and biocompatibility, its use as a drug delivery system for oral administration can be successful. The chosen drug model for this system was melatonin, a well-known compound with multiple beneficial effects in humans. 

Melatonin, known as N-acetyl-5-methoxytryptamine, is a methoxy indole naturally synthesized within pineal cells [13]. It serves various functions, including aiding in the body’s adaptation to the light–dark cycle, mitigating the effects of jet lag, regulating the immune system, and acting as a potent antioxidant agent. Melatonin has also been linked to preserving DNA integrity, suggesting a potential role in cancer prevention as a natural oncostatic agent. Recent developments in the field have suggested that melatonin may find applications in cancer therapy and in managing the adverse effects of anticancer treatment [14]. Typically, the recommended melatonin dosage falls within the range of 0.5 to 10 mg per dose. However, in cases of certain medical conditions, there exists an increased risk of overdose, which can result in patient discomfort. Symptoms of melatonin overdose may include headache, dizziness, nausea, drowsiness, irritability, depression, anxiety, stomach cramps, mild tremors, and low blood pressure, among others. 

Opting for a lower melatonin dosage not only ensures a therapeutic effect sufficient to address the underlying condition but also minimizes the likelihood of adverse side effects and potential harm to vital organs like the liver, kidney, and others by stabilizing the molecule and targeting it at sufficient dose to the site of effect. Another aspect worthy of being accounted for is that MLT is a ubiquitous molecule with natural and powerful antioxidant, anti-inflammatory, and anti-apoptotic properties. Additionally, MLT can diffuse and easily cross all physiological barriers, such as the placenta or the blood–brain barrier (BBB) due to its amphiphilic characteristics. The undoubted efficiency of treatment with MLT has been very much proven, but still, aspects regarding the optimal dose, metabolism, route, and time of administration associated with suitable treatment outcomes remain under discussion [15]. The entrapment of melatonin in biocompatible, non-toxic, and safe delivery systems can prevent its oxidation in circulation, decrease its toxicity, and increase its half-life, resulting in an enhanced pharmacokinetic profile with improved patient compliance [16].

The aim of this study was to create a novel system for encapsulating melatonin within a polymeric carrier. This system was developed using a biocompatible copolymer composed of itaconic acid, acrylic acid, and N-vinylcaprolactam, resulting in micro/nanoparticles that were subsequently coated with chitosan. Comprehensive characterization of these micro/nanoparticles was conducted, including an analysis of their structure and morphology. To assess the delivery capabilities of this system, in vitro and in vivo experiments were performed using a low-dose melatonin-based formulation administered to mice. These experiments involved examining the release profiles, pharmacokinetic and biochemical parameters, and acute systemic toxicity via histopathology, as well as an analysis of the clinical biochemistry of this novel formulation, particularly considering the major organs such as the liver and kidney, following a single oral dose of the formulation containing 180 µg of melatonin. 

## 2. Materials and Methods

### 2.1. Chemicals

In this experiment, various chemicals and reagents were employed. Chitosan of medium molecular weight (CS, Sigma Aldrich product; CAS 9012-76-4; product no 448877) was utilized, along with monomers itaconic acid, acrylic acid, and N-vinyl caprolactam, all obtained from Sigma Aldrich (St. Louis, MO, USA). The initiator system comprised ammonium persulphate (APS, Sigma Aldrich) and 50% hydrogen peroxide solution. The reaction was carried out using ultra-pure distilled water as the solvent. To create the chitosan solution, a 0.5 wt.% acetic acid glacial solution was employed. Purification of the copolymer was achieved using a dialysis bag with a cut-off of 12–14 kDa.

Melatonin of 99% purity, with a molecular weight (Mw) of 232.28 g/mol, was sourced from Alfa Aesar GmbH & Co. KG in Karlsruhe, Germany. All other chemicals and reagents used in this study met analytical grade standards or exceeded them and were procured commercially. Additionally, methanol, dichloromethane (DCM), and sodium acetate buffer solution were employed for HPLC measurements.

### 2.2. Preparation Methods

#### 2.2.1. Synthesis of Copolymer

**Figure 1:**
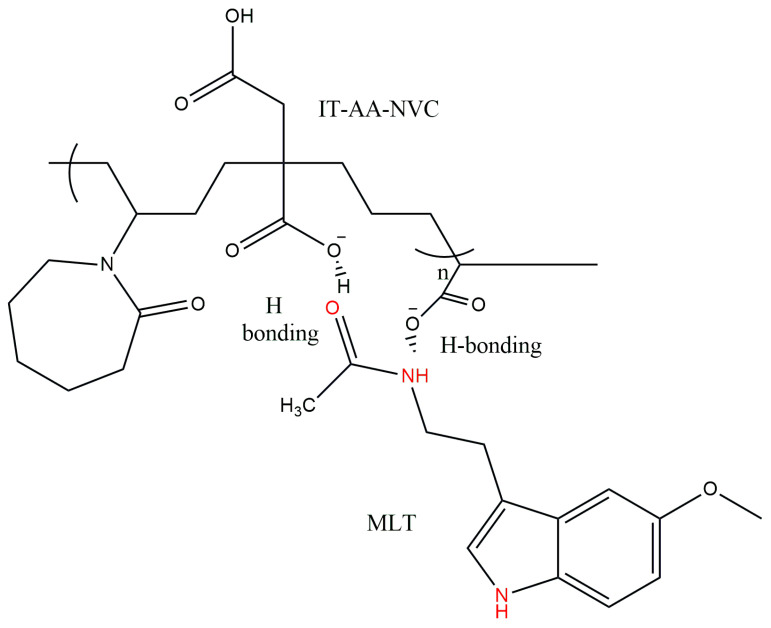
The proposed structure of IT-AA-NVC copolymer and melatonin.

A statistical copolymer composed of acrylic acid (AA), itaconic acid (IT), and N-vinyl caprolactam (NVC) moieties was synthesized by means of a radical polymerization technique. The mole ratio between monomers was 0.046:0.16:0.014 and the total content of monomers was 20 wt%. The batch system comprising the monomers’ mixture, water, and radical initiating system (APS and H_2_O_2_—0.01wt% against monomers) were introduced in a three-neck balloon, which was connected to a condenser to avoid the loss of monomers and water from the system. The radical polymerization reaction was performed at 75 °C for 4 h and then cooled down. The obtained copolymer solution was dialyzed against cold distilled water to remove the unreacted monomers and very-low-molecular-weight specimens by using a dialysis bag with a cut-off of 3.5 kDa. The purification was maintained for 48 h by changing the water periodically. The resulted copolymer (IT-AA-NVC) solution was dried by freeze-dying for 3 days, giving a white powder. The synthesized copolymer was characterized in terms of substance identification by FTIR spectroscopy. The proposed structure was drawn by using CHEMDRAW office software ultra 12.0, and it is presented within Figure 1.

#### 2.2.2. Synthesis of Micro/Nanoparticles

Prior to the particles’ formation, a conjugate of copolymeric carrier and melatonin was prepared by solution-mixing overnight and then lyophilization for a better entrapment of the drug. The incorporation of melatonin into nanoparticles serves as a protective measure, preventing its degradation and enhancing its resistance to photodegradation. This, in turn, facilitates the utilization of therapeutic doses of the molecule [17]. After lyophilization, a white powder-like material was formed (herein noted as PF). Table 1 summarizes the list and the description of the prepared micro/nanoparticles.

The chitosan-coated SE and SM were prepared using a nanoprecipitation technique. For this purpose, samples of the IT-AA-NVC copolymer with and without melatonin (PF and P0) were dissolved in ethanol and then slowly dropped with a micropipette, under strong mixing, into a solution of chitosan 0.5 wt.%, with polyvinyl alcohol solution 5% as colloidal stabilizer. The formed colloidal solution was further mixed for 2 h under continuous stirring. The formed micro/nanoparticles were isolated by centrifugation at 15,000 rpm for 15 min at 6 °C, followed by lyophilization. The obtained powder-like materials were further characterized in their solid and liquid states. 

### 2.3. Characterization Methods

#### 2.3.1. Fourier Transform Infrared Spectroscopy (FTIR) Analysis

The structures of the synthesized polymer and its mixture were confirmed by FT-IR spectra which were recorded with a Perkin-Elmer Spectrum-100 ATR-FTIR instrument (Midland, ON, Canada). The analysis was performed on polymer dried powder obtained by casting the polymeric solution on a Petri dish and subsequent drying at room temperature. The spectrum was obtained after 5 scans between 4000–600 cm^−1^, using reflection on a diamond crystal with an angle of 45° and a resolution of 4 cm^−1^.

#### 2.3.2. Differential Scanning Calorimetry (DSC)

The observed phase-change analyses, such glass transition temperatures DSC analysis, were performed on dried substances (approximatively 5 mg) using a Perkin Elmer Diamond DSC instrument at a heating rate of 10 °C/min under a N_2_ atmosphere from 20 to 250 °C.

#### 2.3.3. Scanning Electron Microscopy (SEM)

The scanning electron microscope (SEM)-based images of the cross-section of the lyophilized formulations were captured using a Carl Zeiss (Jena, Germany) 300 Sigma VP model equipped with Gemini Optical Technology. The magnification is given in the figures.

#### 2.3.4. Analytical Methods for Melatonin Quantification 

The amount of melatonin was analyzed by using an Agilent 1260 high-pressure liquid chromatograph (HPLC) system. The system was equipped with a UV detector and a Chrompack C18 column with a length of 250 * 4.6 mm, Santa Clara, CA, USA. 

For the in vitro simulated conditions, a degassed solution containing 30% water and 70% methanol (*v*/*v* %) was used as the mobile phase. The column’s temperature was set at 45 °C, the flow rate was 1 mL/min, the UV detection was set at 223 nm (specific for melatonin), and the injected volume was 10 µL. For the calibration curve, stock solutions of melatonin were prepared by dissolving 1mg of the pure drug in 100 mL of methanol, and 1 mg/mL stock solution was obtained. Necessary dilutions were made using methanol, and a series of diluted solutions were obtained within a concentration range of 6.25–100 µg/mL. The samples were analyzed, and a calibration line was obtained. The retention time considered for melatonin was set at 5.5 min. The peak area correlated linearly within the concentration range (R2 = 0.9995; y = 77,721x + 57,262), and the concentrations of unknown samples were determined and represented as release profile against time. 

The validation of the method was performed in terms of specificity, stability, accuracy, and precision of the solution. The experiments were carried out in triplicate. The compounds were identified by comparing the retention times of the unknown peaks with the peaks of the reference standards. Limit of detection (LOD) and limit of quantification (LOQ) were determined (LOD—0.945 µg/mL; LOQ—2.86 µg/mL). 

For determination of the drug loading, weighed amounts of lyophilized samples (3 × 1 mg) were placed in a vial, 100 µL of mobile phase was added to each sample, and the matrices were disrupted using an Ultrasonicator (60 kHz × 1 min). To prevent local heating, each sample ‘s disintegration was performed in an ice bath. The samples were then centrifuged at 14,000× *g* for 5 min to remove insoluble polymer fragments. The supernatant was placed in clean tubes and injected into the HPLC. The drug loading (DL) of melatonin was calculated considering the initial amount of melatonin loaded at the beginning of the experiment (0.66% against polymeric matrix). 

The determination of melatonin from plasma as a biological environment (in vivo) was performed by using a different strategy than the HPLC method settled for the in vitro experiments. In particular, the mobile phase consisted of a solution of 85 mM acetic acetate, 0.1 mM EDTA-Na2, and acetonitrile 14% of final volume, pH being adjusted to 4.7. A fluorescence detection (FD) was found to permit the analysis of melatonin without derivatization; this method of detection is more versatile in quantifying melatonin in complex samples (i.e., biological samples). For this purpose, a similar HPLC system was used, but settled for the fluorescence detection at 285/360 excitation/emission wavelengths. 

All analyses were performed at room temperature at a flow rate of 1.0 mL/min. for the calibration curve; human blood samples spiked with various amounts of melatonin were tested using a concentration range of 3.9–120 ng/mL. 

Prior to the analysis of the MLT-spiked blood samples, a two-fold liquid–liquid extraction procedure with chloroform proceeded. Chloroform was selected instead of dichloromethane to avoid any remaining residues that might affect the chromatographic peak of melatonin. 

Various methods have been applied to obtain the optimal conditions for the determination of melatonin from plasma and have failed due to such interreferences with residues from the biological medium. The double extraction method was inspired by the study of Munoz et al. [18]. The procedure applied was as described in the following:

- Both the MLT-spiked blood samples and the ones sampled from the animals were two-fold extracted with chloroform (1:1 *v*/*v*) and mixed by vortex for 1 min each.
- The organic layer was isolated and washed once with a solution of 0.1 N of NaOH, which was further removed by centrifugation.
- The samples were then concentrated by removing the solvent under nitrogen flow until dry.
- The extracted plasma samples for the HPLC measurements were dissolved in 100 µL 0.1 M sodium acetate buffer (pH 4.6), and 10 µL was injected into the HPLC system.
- The calibration curve for the MLT-spiked blood samples was traced and a linear correlation was obtained.
- The concentrations of the unknown blood samples collected from animals were determined by using the equation y = 0.2264x−1.7582, R² = 0.9969. Limit of detection (LOD) and limit of quantification (LOQ) were determined to be 12.078 ng/mL and 36.6 ng/mL, respectively.

#### 2.3.5. In Vitro Release Study of Formulations

The in vitro release profile of melatonin from the synthesized polymeric carrier was simulated by applying a simulated physiological medium (phosphate buffer pH 7.4, T-37 °C). For this purpose, the dialysis-bag diffusion technique was used to study the variation in time of the concentration of melatonin from the micro/nanoparticles. Samples in a dried state were weighted, and then they were placed in the dialysis bag (i.e., cellulose membrane, molecular weight of 12,000–14,000 Da), hermetically sealed, and immersed in 100 mL of phosphate buffer (pH = 7.4). The entire system was kept at 37 ± 0.5 °C, with continuous magnetic stirring at 300 rpm. Samples were periodically withdrawn from the receptor compartment at predetermined time-intervals and the entire receptor phase was replaced with fresh medium at 37 ± 0.5 °C to obtain sink conditions. The amount of the drug that was released out from the polymeric matrix was then determined by the above-described HPLC method for the in vitro conditions. The experiments were carried out in triplicate. The maximum amount of melatonin release, release percent, and half-life (t_1/2_) of the melatonin were calculated. For the data obtained for the first 30 min after the start of the experiment, the Korsmeyer–Peppas model [19,20] was applied to estimate the rate of the drug diffusion and the reaction order. This information was necessary to appreciate the mechanism type of the MLT’s release from the polymeric matrix.

#### 2.3.6. In Vivo Tests

Ethical Approval

- Experimental protocols relevant to the toxicity and biocompatibility studies were implemented according to the institutional recommendations of the Biyotip ve Genom Merkezi (İBG-İzmir) Dokuz Eylül Üniversitesi, Izmir, Türkiye (document no. 1/06.07.2023), Committee for Research and Ethical Issues, in rigorous accordance with international ethical regulations for laboratory animal work [21,22].
- Housing and Care of Animals
  C57BL6 female mice (Control (n:3) and MEL (n:3)) were placed in their cages before the experiment and allowed to adapt to their environment. They were fed ad libitum with standard food and water, adhering to a 12 h light and 12 h dark cycle. The animals were checked regularly every day, and their health was monitored. Beginning 24 h before the experiment, the animals were starved, and 2 h after the drug application, the animals were given food and water again.
- Dosage of Formulations and Their Application to Animals
  While planning the experiments, the human starting dose of 0.5 mg/kg was taken as reference, and the average dose that should be administered to mice was calculated as 1 mg/kg body by allometric scaling. Considering that the amount of melatonin found in formulations is a 0.018 mg MLT/mg formulation, the required amount per kg-bw of each animal was calculated, suspended in 400 µL saline, and applied to the animals by oral gavage.
- Blood Collection for Pharmacokinetic Analyses
  To analyze the amount of melatonin entering the blood following drug administration, blood samples of approximately 30 µL were taken from the retroorbital region of each animal at time points of 0, 30, 45, 60, 75, 90, and 120 min.
- Termination of Experiment and Euthanasia. To investigate whether the formulations had any acute systemic toxic effects, 24 h after drug administration, intracardiac blood samples were taken from the animals under anesthesia, and then the animals were euthanized by cervical dislocation. Blood samples were collected in anticoagulant-free tubes, maintained for 30 min at room temperature for clotting, and then centrifuged to obtain serum samples for biochemical analysis. For histopathological examination, liver, kidney, lung, heart, and brain were separated and fixed in a solution of 10% formalin for 24 h. Then, fixed tissue samples were embedded in paraffin. Sections with sizes of five μm were cut and stained with hematoxylin and eosin (H&E) for the evaluation of histopathological changes [22].
- In vivo release profile. The determination of the amount of melatonin from the plasma was performed by applying the settled HPLC assay used for the biological samples, together with a specifically designed calibration curve. In particular, 10 µL of aliquot was injected and the retention time of melatonin was found to be 9 min. The determinations were performed in three replications. The maximum concentration released within 2 h was determined, together with pharmacokinetic parameters estimated based on the in vivo release profile.

## 3. Results and Discussion

### 3.1. Characterization of Polymeric Matrices

**Figure 2:**
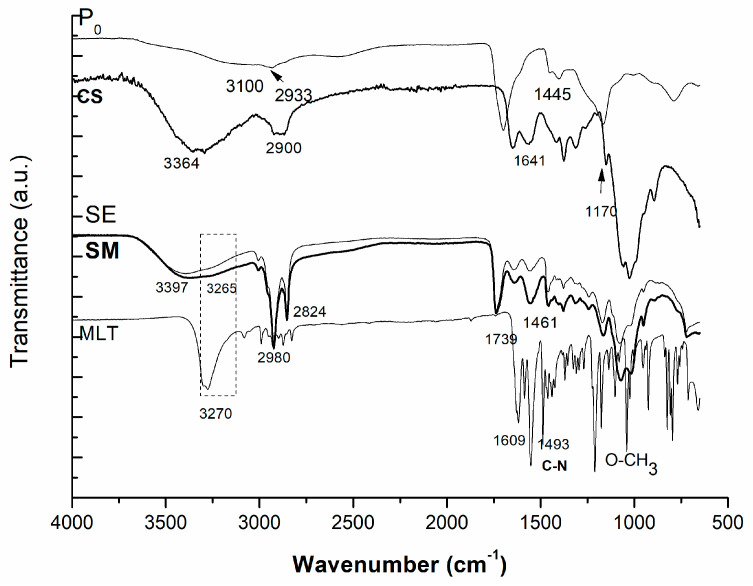
FTIR spectra of the prepared chitosan-coated micro/nanoparticles (SE—without MLT, and SM—with MLT) and pure constituents (P0—pure synthesized copolymer, and CS—pure chitosan) in a dried state.

Polymeric matrices were characterized by means of structure identification (FTIR), thermal characteristics, morphology (SEM observations), and in vitro and in vivo ability to release melatonin. FTIR spectra of prepared samples with and without melatonin are presented within Figure 2.

As can be observed within Figure 2, the characteristic vibration bands of melatonin (MLT), copolymer (P0), and melatonin-loaded polymeric matrices (SM) were determined. Melatonin (MLT), in its pure state, presented vibration bands of amine groups NH at 3300–3500 cm^−1^; NH deformation 1500–1640 cm^−1^; C--O stretching 1600–1680 cm^−1^; C=O 1700 cm^−1^; OH stretching at 3300−3500 cm^−1^; C-O-O-1200–1300 cm^−1^; and O-CH_3_ stretching 1000 cm^−1^.

Chitosan (CS), as coating layer, showed specific vibration bands at 3300–3500 cm^−1^ due to OH and NH groups; 1640 cm^−1^ amide II bands; and 2900 and 2800 cm^−1^ CH_2_ and CH stretching. IT-AA-NVC (P0) copolymer comprises the spectra’s characteristics of the three monomers in their polymerized form: C=O from itaconic acid and acrylic acid; C-N vibration bands of NVC at 1400 cm^−1^; and CH_2_ and CH stretching at 2900 and 2800 cm^−1^. The absence of the vibration band at 1650 cm^−1^ (characteristic of vinyl groups from all three monomers) indicated the success of the polymerization and formation of the copolymer (P0). The spectrum of the final coated polymeric matrix loaded with MLT (SM) presented, as seen within the Figure 1, the vibration peak of chitosan at 3397 cm^−1^, and characteristic vibration bands of OH, NH and intramolecular hydrogen bonds groups; the MLT presence was linked to the appearance of a shoulder at 3200 cm^−1^ within the spectrum of SM. All spectra were interpreted by using available online accepted FTIR spectra databases [23]. Similar outcomes regarding FTIR spectra of the biopolymer were also found in other studies [24].

**Figure 3:**
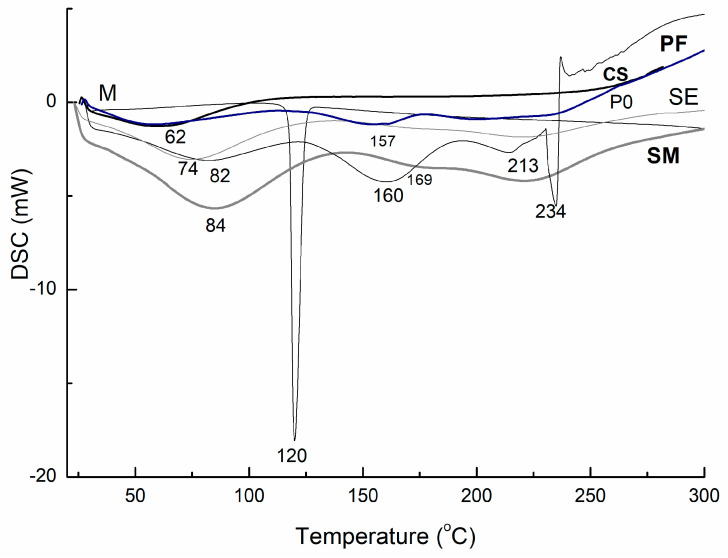
DSC curves of the prepared formulation with (SM) and without MLT (SE) and their pure constituents (chitosan CS, PF polymer IT-AA-NVC with MLT (PF), P0 pure IT-AA-NVC, and M—pure MLT).

The thermal behaviors of polymeric matrices with and without MLT, in a solid state, were determined by means of DSC measurements; the obtained thermograms are presented within Figure 3. 

As represented within Figure 3, pure melatonin showed a melting peak at 120 °C, which is in line with the data reported in the literature [25]. In the thermogram of the copolymeric matrix unloaded (P0) and loaded with melatonin (PF), there were identified endothermic peaks around 157–160 °C and 234 °C; due to the presence of the drug the peaks were shifted to higher temperatures, indicating the interactions between the polymeric matrix and the drug. From the thermograms, it can also be determined that there were thermal events proximately ranging to 100 °C, indicating the loss of water. The amorphous-like structure of pure chitosan (CS) was confirmed by its thermogram being determined as limited to the loss of water at around 60 °C. Conjugates loaded with MLT (PF and SM) showed a melting peak around 230 °C, which is corelated with the melting peak of the drug. The melting peak of around 160 °C, present in all matrices except MLT and CS, indicated the presence of itaconic acid moiety.

**Figure 4:**
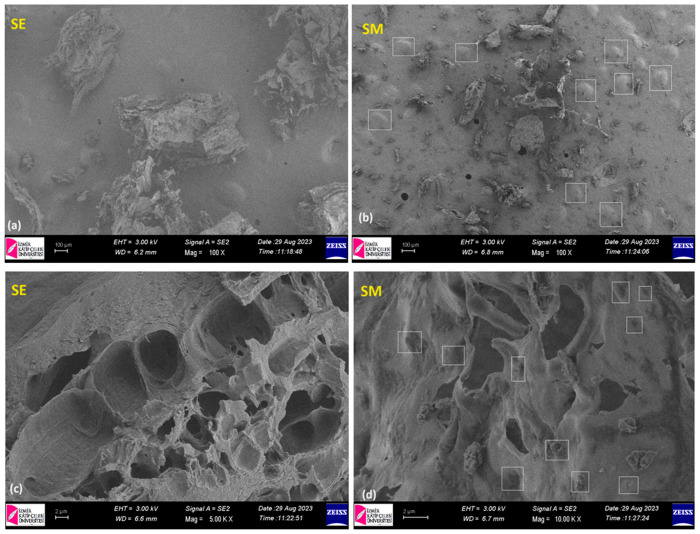
SEM images of the prepared micro/nanoparticles without melatonin (SE) (**a**) magnification 100× (**c**) magnification 5000× and loaded with melatonin (SM) (**b**) magnification 100× and (**d**) magnification 5000×.

The morphology of the prepared micro/nanoparticles was observed by using scanning electron microscopy imaging. SEM images of the unloaded (SE) and loaded micro/nanoparticles (SM) are shown in Figure 4.

As can be observed from Figure 4a,b, a smooth membranous phase consisting of spherical shapes is evident. At higher magnifications, the matrices without MLP exhibited voids, mainly due to the lyophilization. Figure 4c,d show the SEM images of the lyophilized samples with melatonin (SM); these describe the presence of spherical-like particles. The spherical particles are formed in both types of matrices (with and without MLT); the difference is that it seems that the presence of MLT particles inside the polymeric matrix (SM) limited the removal of the frozen water molecules during the lyophilization, so the final particles looked more defined and with an aspect of fullness, MLT playing the role of a filler. The sizes of the pores for SE varied from 1.5 to 7 µm, while for SM, diameters in the range of 1.5 to 4 µm were found. By the loading of the MLT, the visible pore sizes decreased to the point of disappearance, indicating that the loading of drug was successful; thus, there was a good compatibility between the IT-AA-NVC/CS matrix and MLT. Comparable results regarding the morphology of chitosan-based matrices have been reported by other researchers as well [26]. Similarly, this appearance was also observed by Pancescu et al. [27] when studying composite membranes based on chitosan and melatonin, and micro-aggregates of chitosan or melatonin were observed, especially on the surfaces. 

### 3.2. Evaluation of In Vitro and In Vivo Delivery Profile of Melatonin

Prior to the evaluation of the in vitro release behavior of melatonin, the amount of melatonin trapped within the polymeric chains was determined by HPLC assay; it was determined that 1 mg of polymeric micro/nanoparticles contained 180 µg/mL of melatonin. 

**Figure 5:**
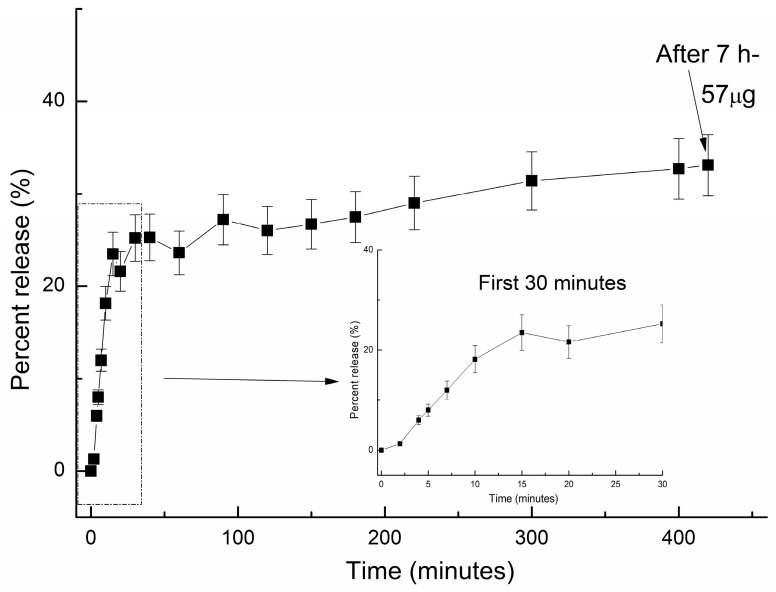
In vitro release profile of melatonin from the polymeric matrix (SM) at 37 °C in pH 7.4 phosphate buffer solution.

Figure 5 describes the in vitro release profile of melatonin within the 7 h from the beginning of the experiment.

As shown within Figure 5, 30 min after the administration of the formulation, a “burst” release was observed, releasing about 26% of the total loaded melatonin. This can be explained by the fact that at the beginning, the release behavior is a swelling-controlled mechanism: the formulation at first becomes swollen and then, once the water has penetrated the macro chains of the polymer, the first-released drug particles are the unbonded or weakly bound ones. The trendline of delivery showed an ascendent shape, indicating that the release is continuing in a sustained manner, reaching a maximum concentration of 47 µg within the first hour, and then a plateau; after 7 h from the administration, about 57 µg (20% more) is released, and the rest of melatonin continues to be released until the polymeric matrix is completely disintegrated. This finding is supported by the theoretical kinetic evaluation of the release profile, which showed an anormal delivery, a matrix-dependent mechanism, and a similarity to the release profiles of other biopolymeric-based drug delivery systems [2,28,29,30].

Most of the studies reporting the in vitro release of melatonin have described a fast release within the first hour and similarly reached a plateau after 4 h, followed by a retarded release of up to 8 h [31]. It has been found that, when comparing the effect of melatonin in prolonged-release dosage form and an immediate-release dosage form, the melatonin in the form of immediate-release was most effective for treating delayed sleep onset, whereas prolonged-release melatonin was more useful for sleep maintenance [32]. Oral absorption of melatonin was reported to be rapid, and peak plasma levels are achieved 20 to 60 min following ingestion. The plasma half-life of melatonin is short, and it is rapidly cleared by the liver [33,34].

Similarly, the half-life of melatonin for the in vitro delivery has been determined to be approximately 20 min. Le et al. [35] mentioned in their report that the half-life of melatonin is between 20 and 50 min, meaning that half of the initial dosage in the body is eliminated after that amount of time. In total, melatonin stays in the human system for about four to five hours due to several factors, such as person’s age, the melatonin dose, and whether it is a fast- or extended-release formulation. Considering data reported in other studies, the release profile was analyzed within this time interval (up to 4–5 h from the administration).

In the evaluation of the kinetics of the in vitro drug release, the Korsmeyer–Peppas semi-empirical formulation described in Equation (1) was applied for the initial release stages (~60% fractional release) [19,20]:

(1) $$\frac{M_{t}}{M_{\infty }}=k_{r\;}t^{n_{r}}$$

where ${M_{t}/M}_{\infty }$ is the fractional drug release, *M*_t_ and $M_{\infty }$ are the cumulative drug release amounts at time *t* and at equilibrium, respectively (or experimental maximum released amount, taken at the plateau of the release curves), *k*_r_ is a rate constant dependent on the characteristics of the drug-loaded system, and *n*_r_ is the diffusional exponent, which defines the type of the release mechanism. A value of *n*_r_~0.5 is characteristic of the Fickian diffusion mechanism of the drug from the polymeric matrix; values in the interval 0.5 < *n*_r_ < 1 are specific to anomalous or non-Fickian behavior. 

A “case II” of the transport mechanism occurs when *n*_r_ = 1, which indicates zero-order kinetics, while a special case II of the transport mechanism is indicated by values of *n*_r_ > 1 [20]. The MLT release profile was plotted as the cumulative percentage of drug amount released versus time. The kinetic parameters for the in vitro profile are summarized within Table 2. As observed, the value of the diffusional exponent, *n*_r_, which characterizes the in vitro drug-release mechanism, was determined to be 0.136, indicating a Fickian diffusion. The small value recorded for the *n*_r_ parameter is correlated with a slow drug-release profile and is caused by a rate of polymer relaxation which is much greater than the rate of drug diffusion; it is also associated with changes in drug solubility. The kinetic parameters determined from the in vitro experiments are summarized within Table 2. Comparable results were reported previously, involving various biopolymer-based matrices [2,28,29,30,36]

**Figure 6:**
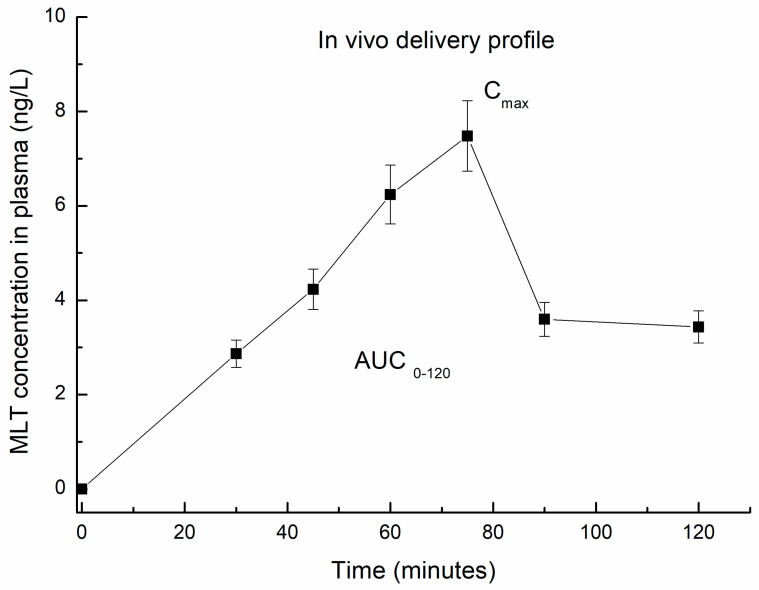
In vivo release profile of melatonin.

As for in vivo measurements, the mean-plasma-melatonin-concentration-versus-time curves after a single oral dose are represented in Figure 6, and mean values of pharmacokinetic parameters (C_max_, t_1/2_, and AUC_0–120h_) are summarized in Table 2. 

Pure melatonin was detected in plasma within the first few hours after its administration in mice. The mean plasma level of melatonin was found to demonstrate a Cmax value of 7.12 µg/mL within 75 min (t_max_), and the elimination half-life (t_1/2_) was determined to be about 14 min, given the kinetics curve profiles and the elimination rate constant (K_el_). The release profile’s curve indicated a fast absorption of melatonin for up to 2 h, until a maximum concentration of melatonin was detected. Subsequently, the elimination curve observed could be described as a first-order process, the amount of drug eliminated being directly proportional to the serum drug concentration. The results obtained were comparable with data reported within other studies [37]. The European Medicines Agency (EMEA) has published a safety assessment and report on the use of Circadin in the form of prolonged-release tablets containing 2 mg of melatonin for oral administration. It reports a low acute toxicological profile by the oral route, with very high values of median lethal dose (LD50), as determined in rodents (1250 mg/kg and >3200 mg/kg respectively in mice and rats) [38].

### 3.3. Acute Systemic Toxicity Evaluation

#### 3.3.1. Biochemical Parameters in Blood

**Figure 7:**
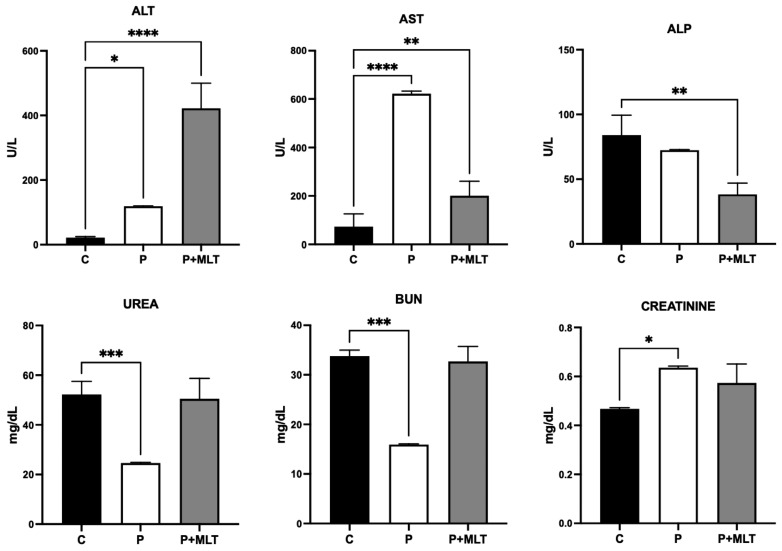
Comparative biochemical parameters obtained from the analyses of the serum samples taken from animals. C, control animals; P, polymer-treated animals; P+MLT, drug (melatonin)-loaded-polymer-treated animals; ALP—alkaline phosphatase; ALT—alanine aminotransferase; AST—aspartate aminotransferase; BUN—blood urea nitrogen; MLT—melatonin. * = *p* < 0.1; ** = *p* < 0.01; *** = *p* < 0.001 and **** = *p* < 0.0001 respectively.

Figure 7 represents the biochemical analyses performed on serum samples obtained from animals. While the levels of ALT, AST, and ALP enzymes were used to evaluate liver functions, BUN, urea, and creatinine in blood were used to evaluate kidney functions.

When evaluating all parameters, it is evident that there is a slight liver toxicity, as indicated by significant changes in blood levels of ALT, AST, and ALP (Figure 7). The polymer alone did not affect blood ALP levels, while the drug-loaded polymer caused a significant decrease in ALP levels (Figure 7, first graph). A decrease in ALP is often due to a lack of zinc, which functions as a cofactor for ALP. It appears that the formulation may cause an acute depletion of zinc in the liver and/or bone marrow. This was accompanied by significant increases in ALT and AST levels. The polymer alone caused an increase in ALT, while drug-loaded polymers more than doubled this increase (Figure 7, second graph). In the case of AST, however, the increases due to the treatments were the opposite; the polymer caused a greater increase compared to the drug-loaded polymer (Figure 7, third graph). The localization of this liver toxicity can be inferred by calculating the ratio (R) of the changed enzyme blood levels. In the present study, the ratio of ALT/ALP of 8.28 strongly suggests that the liver tissue damage is of the hepatocellular type, rather than the cholestatic (R ≤ 2) or mixed-type (2 < R < 5). 

Surprisingly, the polymer alone caused a significant decrease in both urea and BUN levels (Figure 7, first and second graphs, at the lower tier). These two markers indicate kidney dysfunction when elevated in the blood, signifying kidney insufficiency. However, the observed decrease in these parameters may result from either malnutrition (insufficient protein intake) or liver toxicity. Since malnutrition within 24 h, leading to an immediate decrease in protein levels, seems unlikely, liver toxicity is the most probable reason. This is further supported by the direct liver toxicity markers ALP, AS, and ALT. On the other hand, immediate malnutrition cannot be completely ruled out, although it is less likely, as it could also explain the significant decrease in ALP activity in polymer-treated animals. Its zinc-dependent activity was discussed above in the first paragraph.

#### 3.3.2. Histopathologic Examination

Under the current experimental conditions, we have seen adverse tissue changes in treated animals only in the liver and kidney compared to controls. Therefore, only the results of these two organs are presented here, and the others (lungs, heart, and brain) are omitted. 

**Figure 8:**
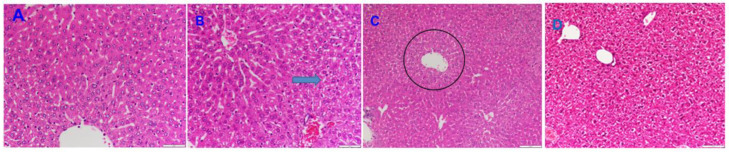
The representative microscopy images of the histopathology results for the liver tissue. (**A**) shows the liver section of one of the healthy control animals; (**B**) shows the accumulation of microlipid droplets in hepatocytes (arrow) in one of the drug-loaded-polymer-treated animals; (**C**) shows the slight degeneration in hepatocytes (the circle indicates that the degeneration that occurs is due to chemical-induced damage in the hepatocytes located around the central vein of the drug-loaded-polymer-treated animal); (**D**) shows the liver section of one of the polymer-treated animals. Magnification 100×.

Figure 8 presents the histopathological results for the liver. As marked with the arrow, an accumulation of microlipid droplets in hepatocytes and a slight degeneration in hepatocytes were observed.

**Figure 9:**
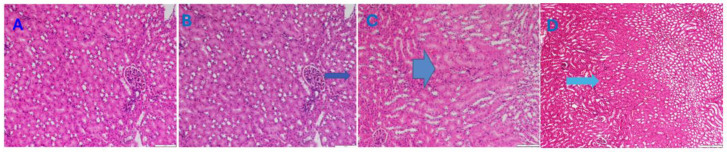
The representative microscopy images of the histopathology results for the kidney tissue. (**A**) represents a kidney section of one of the healthy control animals. (**B**) shows a proximal tubular epithelium, homogeneously swollen cytoplasm, and corresponding narrowing of the lumen in one of the drug-loaded-polymer-treated animals. (**C**) represents a section of the kidney with swollen proximal tubular epithelium at the corticomedullary border, again, one of the drug-loaded-polymer-treated animals. (**D**) shows the kidney section of one of the polymer-treated animals. According to the results obtained, it seems that the polymer treatment caused a slight swelling in the tubulus and a narrowing in the lumens (Figure 9D, arrow), while the medullary tubules were normal. The cytosol of the proximal tubule epithelial cells showed homogeneous swelling, and their lumens showed a corresponding narrowing. However, clinical biochemistry of the kidney was not in accordance with a theory of corresponding damage, according to either plasma creatinine, urea, or blood urea nitrogen (BUN). Magnification 100×.

The histopathology of one of the treated animal’s kidneys can be seen in Figure 9. Similarly to the findings for the liver, there were slight changes in the kidney under these experimental conditions. 

The polymeric matrices without melatonin were tested as well to assess the potential toxic characteristics of the polymeric matrix shown in previous studies involving chitosan, alginate, and poly (N vinyl acrylamide) [2,28,29,30]. Even though it seemed initially that polymer treatment caused a slight swelling in tubules and an increase in ALT, the effect of polymer on ALP was insignificant. Additionally, Hui et al. [39] recently reported comprehensive information on the toxicity of chitosan-based nano/microparticles, as well as conclusions as to their suitability for use as a carrier for lipophilic drugs, e.g., methotrexate, tamoxifen, etc. These hypotheses can support future approaches aiming to reduce animal testing, allowing the number of experimental animals to be reduced, as well as the dose and testing time, in line with the enforced OECD guidelines regarding toxicity testing. 

## 4. Conclusions

A new polymer based on natural and biocompatible monomers was synthesized, namely, IT-AA-NVC copolymer, and it was further formulated as micro/nanoparticles by coating with chitosan (CS) solution in the presence of PVA as stabilizer. Melatonin (MLT), reported as being a lipophilic drug, is secreted by the pineal gland, and mainly helps in the body’s adaptation to the light–dark cycle. Although it brings many benefits to the human body, an overdose can bring discomfort to patients, as evidenced by adverse outcomes in the literature. Therefore, encapsulation in a polymeric carrier can significantly reduce many disadvantages, both by stabilizing the molecule and by providing a sustained release. The entrapment of melatonin within the polymeric chains of IT-AA-NVC copolymer and chitosan was confirmed by SEM images and the amount entrapped was quantified by HPLC analysis. In particular, 27% of the initially loaded melatonin was encapsulated/trapped within the polymeric matrix via electrostatic and hydrogen bonds between the OH, NH and COOH groups of both the copolymer and the melatonin. 

The in vitro release profile of melatonin from the polymeric matrix seemed to follow a Fickian diffusion behavior, with up to 57 µg/mL of melatonin being released within 7 h, about 32% of the total loaded amount (i.e., 180 µg/1 mg formulation); the trendline suggests that the rest of melatonin will slowly be released as the polymeric matrix erodes. 

Biochemical analyses of the blood samples of mice in the control and treatment groups (polymer, and polymer loaded with MLT) showed a slight liver toxicity, as indicated by changes in blood levels of ALT, AST, and ALP. The polymer alone did not affect blood ALP levels, while the drug-loaded polymer caused a significant decrease in ALP levels, mainly due to a lack of zinc, which functions as a cofactor for ALP. It seems that the formulation may cause an acute depletion of zinc in the liver and/or bone marrow, accompanied by significant increases in ALT and AST levels. However, the effect of polymer on ALP can be considered to be insignificant. This type of adversity is generally seen in the case of numerous approved drugs as well, in a transient mode. Therefore, it can be interpreted as tolerable [40,41,42]. Any indication of cholestatic or mixed-type adversity might have been more alarming. Nevertheless, repeated dose toxicity profile for both polymer and drug-loaded polymer should be obtained in future studies, a project which is already planned by our group.

Histopathology findings for liver samples were largely in accordance with clinical biochemical results even though there were slight adverse changes at the hepatocellular level. 

Considering the kidney, the results for clinical biochemistry and histopathology were not consistent; there were, again, slightly adverse changes in the histopathology of the kidneys, while neither blood urea BUN nor creatinine showed a significant change between the control and treatment groups. 

It should be kept in mind, however, that this toxicological evaluation was performed only after a single dose and within 24 h, namely, in acute mode. Since drug-induced liver injury (DILI) is the major reason behind drug attrition and withdrawal, the safety of the current formulation should also be evaluated chronically; this is currently being investigated in a further study ongoing in our laboratory. 

## Figures and Tables

**Table 1:** List and description of the prepared micro/nanoparticles.

Code	Description
P0	IT-AA-NVC copolymer
PF	IT-AA-NVC copolymer loaded with melatonin
SE	Micro/Nanoparticles of IT-AA-NVC coated with chitosan
SM	Nanoparticle of IT-AA-NVC/Melatonin coated with chitosan
MLT	Melatonin
CS	Chitosan

**Table 2:** Pharmaco-kinetic parameters determined from the in vivo and in vitro release profiles of melatonin from the IT-AA-NVC/ CS-based polymeric matrix.

Pharmacokinetic/In Vitro Kinetic Parameter	In Vivo	In Vitro
Dose	1 mg/kg BW	10 mg
C_max_	7.15271 µg/mL	47 µg/mL
t_max_	75 min	40 min
k_r_	-	1 (min)^−n^
n_r_	-	0.137
R^2^n_r_/R^2^k_r_	-	0.99
AUC_0→120_	450.9 µg.min/mL	-
K_el_	4.8835 h^−1^	8.14 h^−1^
t_1/2_	14 min *	20

* t_1/2_ was determined by applying the equation t_½_ = 0.693/K_el_; K_el_—the elimination rate constant (Kel) represents the fraction of drug eliminated per unit of time; n_r_—diffusional exponent that characterizes the in vitro drug-release mechanism; k_r_—release kinetic constant; R^2^n_r_/R^2^k_r_—correlation coefficient.

## Data Availability

Data is contained within the article.
